# Seasonal dynamics and prognostic determinants of respiratory failure in critically ill adults: a multicenter retrospective observational study in Northern China

**DOI:** 10.1186/s12890-026-04576-6

**Published:** 2026-08-18

**Authors:** Dong Miao, Xiaolei Ye, Li’ao Wang, Xinfeng Bai, Meiying Wang, Yunsheng Hou, Zhiyong Song, Jun Gan, Guojing Bao, Jingjing Yu, Junfeng Zheng, Yingping Tian

**Affiliations:** 1 Department of Emergency, The 980th Hospital of the PLA Joint Logistics Support Force (Primary Bethune International Peace Hospital of PLA), Shijiazhuang, 050082 China; 2 The Center for Disease Prevention and Control in Western Theater Command of PLA Joint Logistic Support Force, Lanzhou, 730030 China; 3 Hebei Children’s Hospital of Hebei Medical University, Shijiazhuang, 050031 China; 4 The Second Hospital of Hebei Medical University, Shijiazhuang, 050000 China

**Keywords:** Respiratory failure, Critically ill patients, Meteorological factors, Seasonal variation, Risk factors

## Abstract

**Background:**

Respiratory failure is a common and severe syndrome in critical care, associated with poor prognosis, and its incidence and healthcare burden among critically ill patients are increasing. This study aimed to investigate seasonal patterns of respiratory failure admissions and their associations with meteorological factors and to evaluate the contributions of clinical and meteorological features to model-predicted in-hospital mortality.

**Methods:**

This multicenter retrospective observational study included adults with respiratory failure admitted to emergency intensive care units (EICUs) between 2022 and 2024. Clinical and meteorological datasets were linked based on admission time and regional location. Seasonal trends were analyzed using seasonal-trend decomposition with Loess (STL), while quasi-poisson generalized additive models (QP-GAMs) and distributed lag non-linear models (DLNMs) were used to examine meteorological effects. A random forest model combined with SHapley Additive exPlanations (SHAP) was used to quantify global and individual feature contributions to model-predicted in-hospital mortality.

**Results:**

Among 1,837 critically ill patients with respiratory failure, 57.8% were male, with a median age of 66 years; 34.2% had hypoxemic type and 64.1% hypercapnic type, with overall in-hospital mortality of 54.3%. STL analysis revealed marked seasonal variation, peaking in winter and reaching a minimum in summer. QP-GAMs and DLNMs suggested nonlinear and lagged associations of temperature and relative humidity with monthly respiratory failure admission counts. In the random forest model, age and mechanical ventilation had the largest mean absolute SHAP values, followed by other clinical features.

**Conclusion:**

This study revealed nonlinear and lagged associations between meteorological factors and monthly respiratory failure admissions, with the highest admission counts observed in winter, followed by spring. In the fitted random forest model, clinical and physiological features contributed more to mortality predictions than meteorological variables.

**Supplementary Information:**

The online version contains supplementary material available at https://doi.org/10.1186/s12890-026-04576-6.

## Introduction

Respiratory failure is a common and severe clinical syndrome characterized by the inability of the respiratory system to maintain adequate oxygenation (arterial partial pressure of oxygen to fraction of inspired oxygen ratio [PaO₂/FiO₂] ≤ 300 mmHg) or effectively eliminate carbon dioxide (arterial partial pressure of carbon dioxide [PaCO₂] ≥ 50 mmHg with pH < 7.35), regardless of whether it occurs acutely, chronically, or as an acute-on-chronic exacerbation, and independent of underlying etiology [1]. Rather than representing a single disease, respiratory failure can arise from heterogeneous and sometimes overlapping conditions, including severe pneumonia and other infections, acute exacerbations of chronic respiratory diseases—particularly chronic obstructive pulmonary disease—and acute heart failure with cardiogenic pulmonary edema [2]. Its occurrence often necessitates advanced respiratory support, continuous monitoring, and multi-organ management, and is associated with high mortality and substantial healthcare resource utilization. In recent years, with population aging and the increasing prevalence of chronic comorbidities, the number of patients with respiratory failure admitted to emergency intensive care units (EICUs) has been rising [3–5]. However, existing studies have primarily focused on specific disease subgroups or single-center cohorts, and systematic, comprehensive descriptions of adult patients with respiratory failure in EICUs are still lacking [6, 7].

Meteorological factors such as temperature, humidity, and precipitation may influence respiratory disease occurrence, but these associations are not uniform across etiologies. Recent studies have reported disease-specific seasonal and environmental patterns in pneumonia and chronic respiratory diseases, including asthma and chronic obstructive pulmonary disease [8, 9]. Heart failure hospitalizations also show seasonal variation, with a winter peak in admissions but a different temporal pattern for in-hospital mortality [10]. Shijiazhuang lies in the North China Plain and has a sub-humid warm-temperate continental monsoon climate with marked seasonal variation; a local meteorological study reported a mean daily temperature of 14.5 °C and mean relative humidity of 57.4% [11]. Thus, the seasonality observed for all-cause respiratory failure may partly reflect changes in the underlying etiologic composition and should not be assumed to apply uniformly to each cause. Nevertheless, ecological studies focusing on critically ill patients with respiratory failure remain scarce. Existing research has rarely integrated individual-level clinical data with regional meteorological indicators, limiting comprehensive assessment of environmental impacts on the development of respiratory failure [12]. In addition, seasonal variations in meteorological conditions may affect respiratory function and patient susceptibility through multiple pathways, yet these mechanisms have not been fully explored in the population of critically ill patients admitted to EICUs.

Established intensive care unit (ICU) scoring systems serve different purposes. The Sequential Organ Failure Assessment (SOFA) score provides a repeatable assessment of dysfunction across six organ systems, whereas the Acute Physiology and Chronic Health Evaluation (APACHE) and Simplified Acute Physiology Score (SAPS) systems were developed primarily to estimate mortality risk [13]. Compared with these predefined scoring systems, machine-learning approaches can capture nonlinear relationships and interactions among predictors and, when combined with SHapley Additive exPlanations (SHAP), provide feature-level interpretations; however, their clinical utility depends on adequate calibration, external validation, and interpretability [14]. Therefore, interpretable machine-learning approaches should be regarded as complementary to, rather than replacements for, established ICU scoring systems.

To address these research gaps, this study focused on adult critically ill patients with respiratory failure admitted to EICUs. We established a multicenter retrospective observational database, integrating patient demographic information, clinical characteristics, mechanical ventilation status, and outcomes with regional meteorological data. The study aimed to describe the clinical epidemiology of critically ill patients with respiratory failure, investigate seasonal trends and their associations with meteorological factors, and identify key risk factors influencing in-hospital mortality, thereby providing a scientific basis for early clinical recognition and management.

## Methods

### Study population

This multicenter retrospective observational study was led by a tertiary teaching hospital in Shijiazhuang, China, and conducted in the EICUs of two hospitals in the city. The study period spanned from January 1, 2022, to December 31, 2024. All adult patients who developed respiratory failure during EICU admission within this period were included. For patients with multiple admissions during the study period, only the first admission was considered. Patients directly transferred from other hospitals were included if their initial EICU diagnosis met the criteria below. Inclusion criteria were: (1) age ≥ 18 years; (2) presence of respiratory failure on admission, defined as arterial blood gas abnormalities (PaO₂ < 60mmHg or PaCO₂ ≥ 50mmHg with pH < 7.35 under room air) or the need for invasive or non-invasive mechanical ventilation due to respiratory failure [15, 16]; (3) complete case data, including demographic information, admission diagnosis, mechanical ventilation status, and outcomes. Exclusion criteria were: (1) transfer to other departments within 24 h of admission; (2) pregnancy or postpartum status; (3) missing data ≥ 30% of key clinical variables.

### Data sources and variable definitions

Clinical data were obtained from the medical record archives or electronic health record (EHR) systems of the EICUs at the two participating hospitals. Data were extracted on a per-patient per-admission basis and manually entered into the study database, including admission date, demographic characteristics, respiratory failure phenotype, vital signs, Charlson Comorbidity Index (CCI), mechanical ventilation status, and outcomes. Respiratory failure was classified according to physiological phenotype as hypoxemic, hypercapnic, or mixed. The database did not contain a prespecified variable assigning a single primary etiology of respiratory failure; therefore, etiology-based subgroup analyses comparing pneumonia with non-infectious causes were not performed. Monthly meteorological data from all stations in Shijiazhuang between January 2022 and December 2024 (36 months) were obtained from the National Meteorological Science Data Center (http://data.cma.cn). The meteorological variables included in this study were mean temperature, mean maximum temperature, mean minimum temperature, mean precipitation, and mean relative humidity. Complete and consistently measured district-level air-pollution data that could be matched to both hospital areas throughout the entire study period were unavailable. Therefore, ambient air pollutants were not included in the analyses, and the estimated associations with meteorological factors were not adjusted for air pollution. To reflect patients’ residential environment and climate exposure, each admission was matched with the meteorological data of the corresponding hospital’s district, representing the environmental exposure for patients in that hospital. For overall analyses, the mean values of meteorological indicators from the two hospital districts were used as regional-level macro-environmental exposures. Derived variables included respiratory failure phenotypes (hypoxemic, hypercapnic, and mixed) [17] and seasonal groups (spring: March-May; summer: June-August; autumn: September-November; winter: December-February) [18]. In ecological analyses, monthly counts of respiratory failure admissions were matched with meteorological data, whereas individual-level regression analyses incorporated the meteorological indicators corresponding to each patient’s admission month.

### Data quality control

All cases were confirmed by attending physicians or higher-level clinicians based on medical records, and relevant clinical data were manually collected. Data extraction was independently performed by two analysts, and discrepancies were resolved through discussion. Variables with ≥ 30% missing values were assessed for their importance to the outcomes before deciding whether to retain them. For variables with < 30% missing values, missing data were imputed using the multiple imputation by chained equations (MICE) method [19]. Variable-specific pre-imputation missingness and corresponding handling are summarized in Supplementary Table 1.

### Statistical analysis

Monthly counts of critically ill patients with respiratory failure were constructed as a univariate time series to systematically assess the temporal dynamics of disease occurrence. The series was decomposed into long-term trend, seasonal, and residual components using seasonal-trend decomposition based on locally estimated scatterplot smoothing (STL) [20]. This method was applied to robustly handle non-stationary epidemiological data and isolate true seasonal cycles from underlying trends. The long-term trend reflects the overall evolution of case numbers over time, the seasonal component captures cyclical fluctuations across months, and the residuals represent random variations not explained by trend or seasonality. To quantitatively describe seasonal effects, a seasonal index for each month was calculated as the ratio of monthly case counts to the annual mean, indicating the relative risk of respiratory failure admission in that month. A seasonal index greater than 1 denotes a higher-than-mean monthly case count, suggesting a potential high-risk period, whereas an index less than 1 indicates a lower-than-mean monthly case count.

Mean temperature, maximum temperature, minimum temperature, precipitation, and mean relative humidity were matched with the monthly counts of critically ill patients with respiratory failure. The time-shift approach was used to calculate Pearson correlation coefficients between meteorological variables and case numbers, with lags ranging from − 2 to + 2 months to assess delayed effects. Quasi-Poisson generalized additive models (QP-GAMs) were employed to model the nonlinear relationships between meteorological factors and monthly case counts [21]. The Quasi-Poisson family was specifically utilized to account for the overdispersion of clinical count data, while the generalized additive model (GAM) framework allowed for flexible fitting of non-linear exposure-response curves [22]. Long-term trends and smooth seasonal terms were incorporated into the models to control for temporal trends and seasonal fluctuations. Furthermore, distributed lag nonlinear models (DLNMs) were applied to evaluate the lagged and cumulative effects of mean temperature and mean relative humidity [23], with a maximum lag of 2 months. DLNMs provided a unified framework to simultaneously quantify non-linear and delayed cumulative effects, overcoming the collinearity issues common in lagged environmental exposures [24].

Based on the collected clinical and meteorological data, a random forest model was constructed to identify predictors of patient mortality. The random forest algorithm was used to capture complex and nonlinear relationships among the included features [25]. The following 17 variables were included as predictors: age, sex, body mass index, respiratory failure phenotype, heart rate, noninvasive systolic blood pressure, noninvasive diastolic blood pressure, respiratory rate, oxygen saturation, body temperature, mechanical ventilation status, season of admission, mean temperature, mean minimum temperature, mean maximum temperature, mean relative humidity, and mean precipitation. In-hospital mortality was specified as the outcome. Categorical variables were label-encoded, and the dataset was randomly split into training and testing sets at a 7:3 ratio using a random seed of 42. The model was fitted in the training set using 500 classification trees. No preliminary feature selection or hyperparameter optimization was performed. After model fitting, TreeExplainer was used to calculate SHapley Additive exPlanations (SHAP) values for the in-hospital death class in the testing set, enabling both global feature ranking and individual-level interpretation [26, 27]. Global feature importance was determined by ranking the mean absolute SHAP values across testing-set observations, and a bar plot of the top 15 variables was generated to illustrate overall contributions. For individual-level interpretation, the 10 features with the largest absolute SHAP values for one testing-set sample were displayed in a SHAP waterfall plot, providing an intuitive depiction of key factors contributing to the individual model prediction.

Univariable and multivariable logistic regression analyses were performed to assess the association between the CCI and in-hospital mortality. The multivariable model was adjusted for age, sex, respiratory failure phenotype, and mechanical ventilation status, and the results are reported as odds ratios (ORs) with 95% confidence intervals (CIs).

All statistical analyses and graphical presentations were performed using R version 4.3.1, Python version 3.10 and GraphPad Prism version 9.5. A two-sided *P* value of < 0.05 was considered statistically significant. Continuous variables were summarized as mean±standard deviation (x̄±s) or median and interquartile range (IQR), and categorical variables were summarized as frequencies and percentages (*n*, %).

## Results

### Characteristics of the baseline population

After applying the inclusion and exclusion criteria, a total of 1,837 patients with respiratory failure admitted to the EICUs of two collaborating hospitals between January 1, 2022, and December 31, 2024, were included in the final analysis. The median age was 66 years (IQR, 56–77), and 57.76% were male. Hypoxemic respiratory failure accounted for 34.19%, hypercapnic respiratory failure for 64.07%, and mixed-type for 1.74%. A total of 98.75% of patients received mechanical ventilation, including both invasive and non-invasive modalities. The seasonal distribution of admissions peaked in winter, followed by spring and autumn, with the lowest in summer. The overall in-hospital mortality rate was 54.33% (Table 1). The median CCI was 5 (IQR, 3–8) (Table 1). In logistic regression analysis, each one-point increase in CCI was associated with higher odds of in-hospital mortality in both the univariable model (OR, 1.30; 95% CI, 1.26–1.35; *P* < 0.001) and the multivariable model adjusted for age, sex, respiratory failure phenotype, and mechanical ventilation (adjusted OR, 1.26; 95% CI, 1.20–1.32; *P* < 0.001) (Supplementary Table 2).

**Table 1:** Overview of annual statistics for EICU patients with respiratory failure

	Total	Year		
		2022	2023	2024
No. of cases	1837	634	592	611
No. of death (%)	998 (54.33)	339 (53.47)	319 (53.89)	340 (55.65)
Age, median (IQR)	66 (56–77)	66 (56–77)	67 (55–76)	66 (57–78)
Charlson Comorbidity Index, median (IQR)	5 (3–8)	5 (3–7)	5 (3–8)	6 (3–8)
No. (%) of male cases	1061 (57.76)	370 (58.36)	345 (58.28)	346 (56.63)
No. (%) of cases by respiratory failure type				
Hypoxemic	628 (34.19)	230 (36.28)	199 (33.61)	199 (32.57)
Hypercapnic	1177 (64.07)	390 (61.51)	383 (64.70)	404 (66.12)
Mixed	32 (1.74)	14 (2.21)	10 (1.69)	8 (1.31)
No. (%) of cases on mechanical ventilation	1814 (98.75)	629 (99.21)	578 (97.64)	607 (99.35)
No. (%) of cases by season				
Spring	479 (26.08)	160 (25.24)	155 (26.18)	164 (26.84)
Summer	384 (20.90)	140 (22.08)	122 (20.61)	122 (19.97)
Autumn	440 (23.95)	153 (24.13)	143 (24.16)	144 (23.57)
Winter	534 (29.07)	181 (28.55)	172 (29.05)	181 (29.62)

*Abbreviations*: *EICU* emergency intensive care unit, *IQR* interquartile range

### Temporal trends and seasonality

**Fig. 1:**
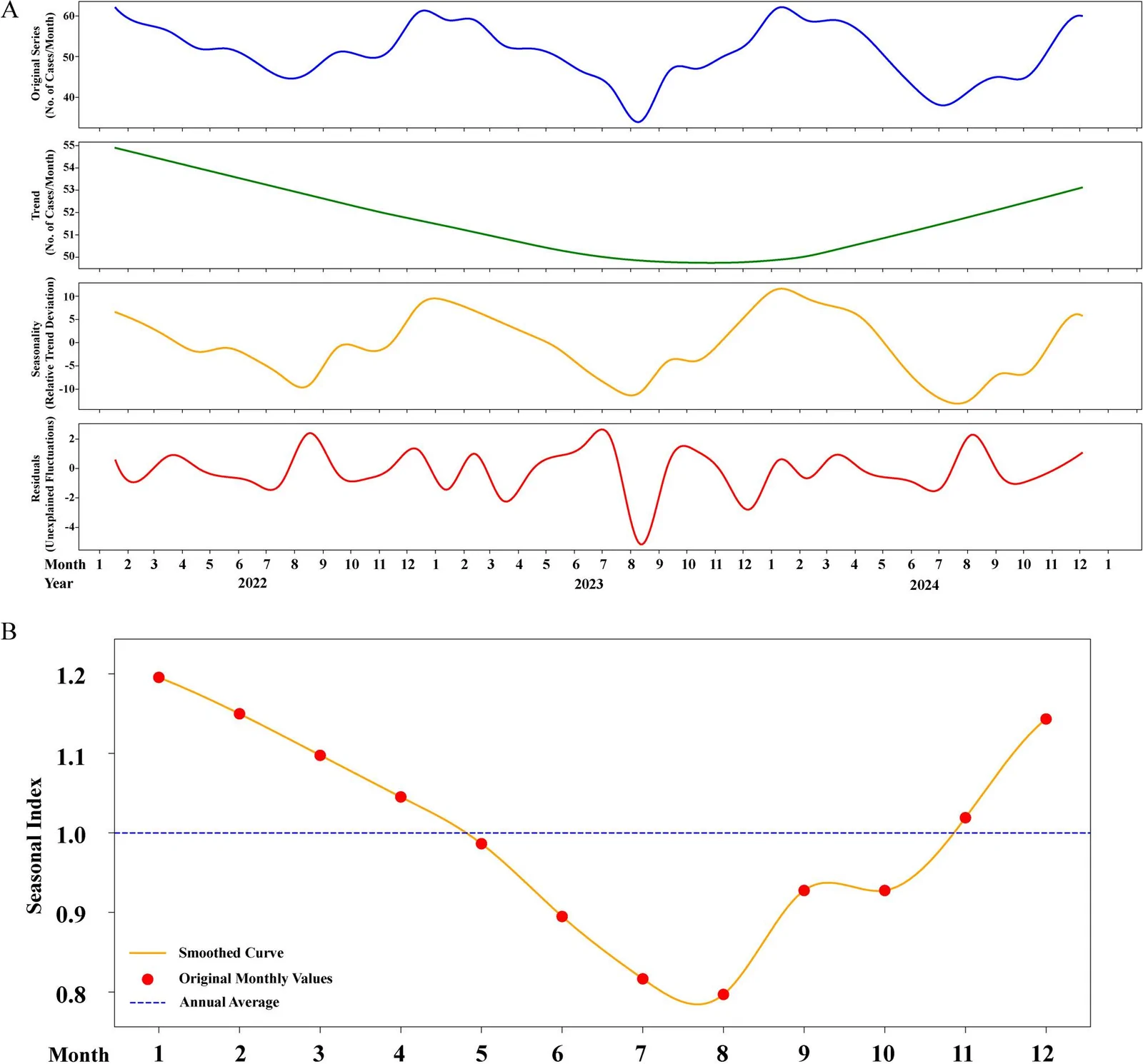
Monthly time trends and seasonal patterns of critically ill patients with respiratory failure. **A** Monthly case counts and long-term trend; **B** Seasonal index

During the 36-month study period, the monthly admissions of patients with respiratory failure to the EICUs ranged from 34 to 62, with an overall pattern of decline followed by a subsequent increase (Fig. 1A). STL further separated the time series into three components: the long-term trend indicated a gradual increase in case numbers in the latter part of the study; the seasonal component revealed a clear cyclical pattern, with peaks in winter (December-February) and troughs in summer (June-August); and the residuals showed limited fluctuation, indicating a good model fit. Analysis based on seasonal indices (Fig. 1B) showed that the relative risk of admission was highest in January (index 1.20), approximately 20% above the annual mean, and lowest in August (index 0.80), about 20% below the annual mean. Overall, admissions for respiratory failure exhibited both long-term dynamics and pronounced seasonal variation, with winter representing the high-risk period and summer the low-risk period.

## Association analysis of meteorological factors

**Fig. 2:**
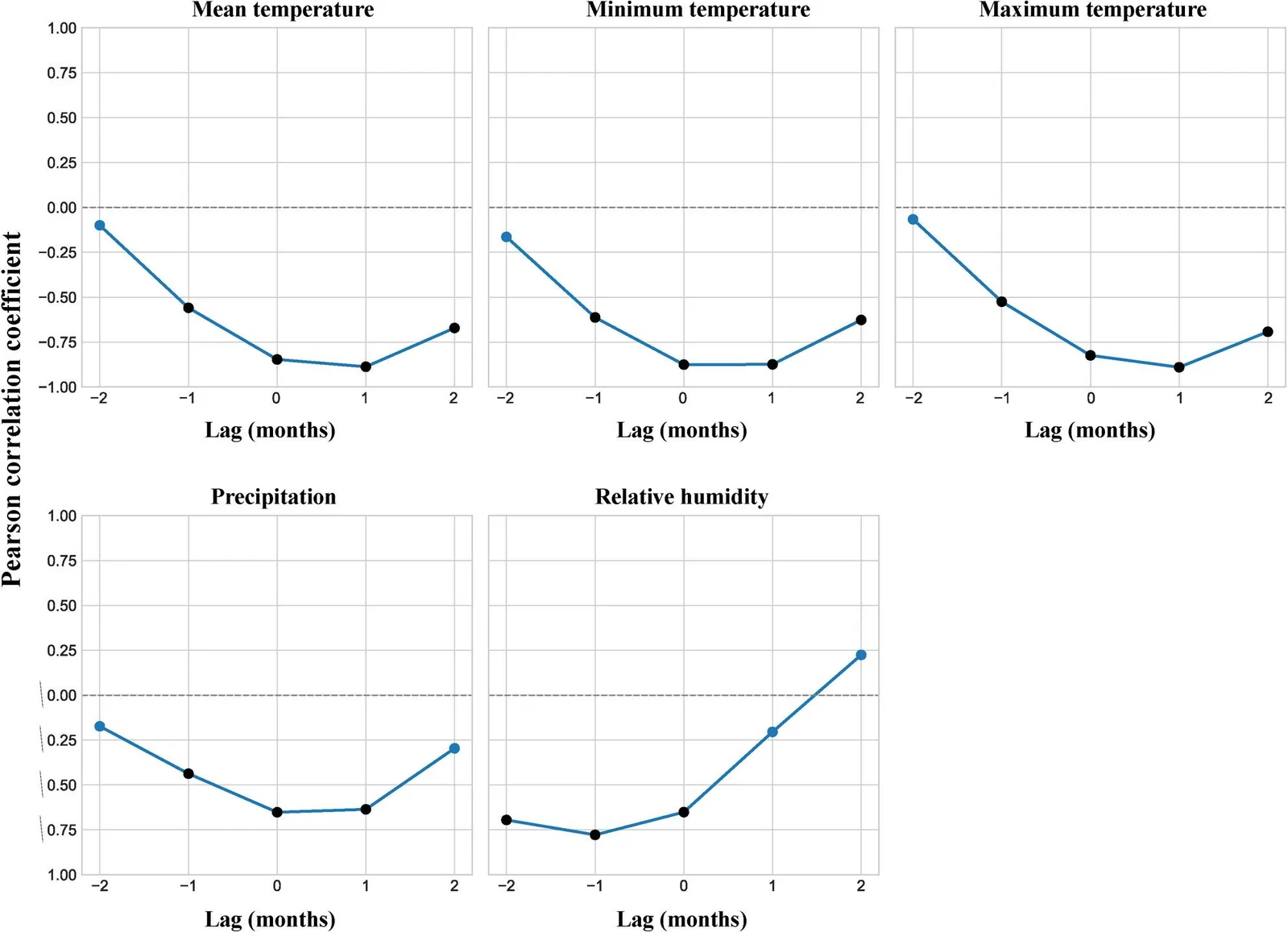
Lagged correlations between meteorological variables and monthly number of critically ill patients with respiratory failure

To clarify the baseline climatic characteristics and the temporal relationships among different meteorological variables, the mean monthly values of temperature, precipitation, and relative humidity averaged over the three-year study period (January to December) are presented in Supplementary Figure S1. The region exhibits a typical temperate monsoon climate, with temperature and precipitation showing highly synchronized seasonal peaks during the summer months. Lagged correlation analysis showed that meteorological variables exerted delayed effects on the monthly number of critically ill patients with respiratory failure (Fig. 2). The mean temperature exhibited the strongest correlation at a 1-month lag (*r*=-0.89, *p* < 0.05), indicating that changes in temperature affected case numbers with a one-month delay. Similar patterns were observed for both the mean minimum and maximum temperatures, which reached their peak correlations at a 1-month lag. Mean precipitation was negatively correlated with case numbers, with the strongest correlation observed at a 0-month lag (*r*=-0.65, *p* < 0.05). The correlation between mean relative humidity and case numbers followed a bidirectional pattern (i.e., a U-shaped relationship where both abnormally low and excessively high relative humidity levels were associated with an increase in case numbers), with the strongest correlation occurring at a −1-month lag (*r*=-0.78, *p* < 0.05). Supplementary Table 3 presents the Pearson correlation coefficients and corresponding p-values for each meteorological variable at lags ranging from −2 to +2 months.

**Fig. 3:**
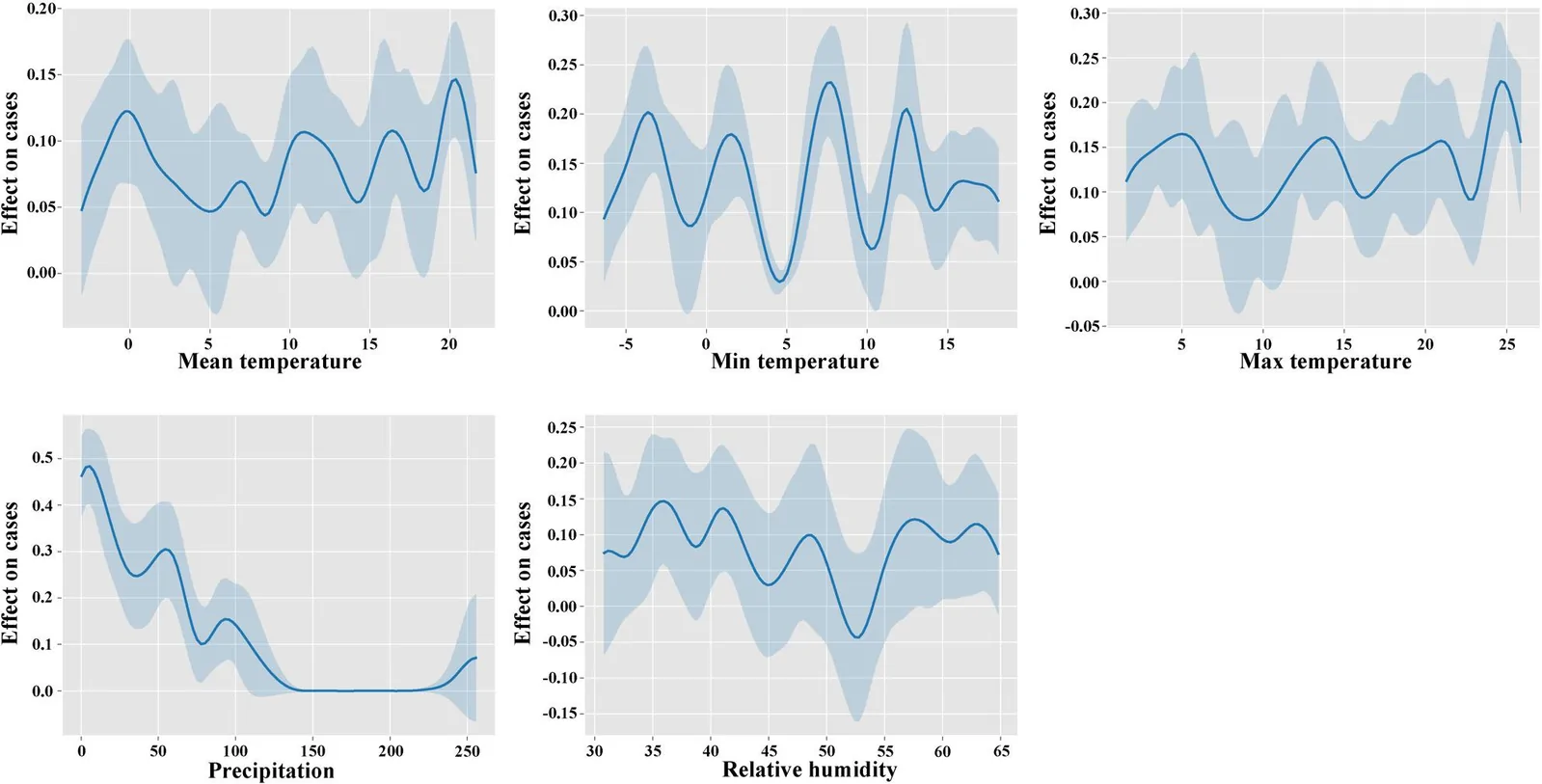
Partial effects of meteorological factors on monthly case counts. Generalized additive models (GAMs) were used to fit the nonlinear relationships between mean temperature, minimum temperature, maximum temperature, mean precipitation, and mean relative humidity with monthly case numbers. The blue lines represent the estimated partial effects, and the light blue shaded areas indicate the 95% confidence intervals

GAM analysis indicated that mean temperature, mean minimum temperature, and mean maximum temperature were all positively and nonlinearly associated with the number of cases. The effects were most pronounced at approximately 0 °C and 20 °C for mean temperature, around 7.5 °C and 12 °C for mean minimum temperature, and near 24 °C for mean maximum temperature. Mean precipitation was negatively associated with case counts, with the effect plateauing when precipitation exceeded 120 mm. Relative humidity exhibited a bidirectional nonlinear relationship with case numbers, with increased risk observed when humidity was below 51% or above 54%. The 95% confidence intervals suggested that these effects were statistically significant across most of the observed ranges (Fig. 3).

**Fig. 4:**
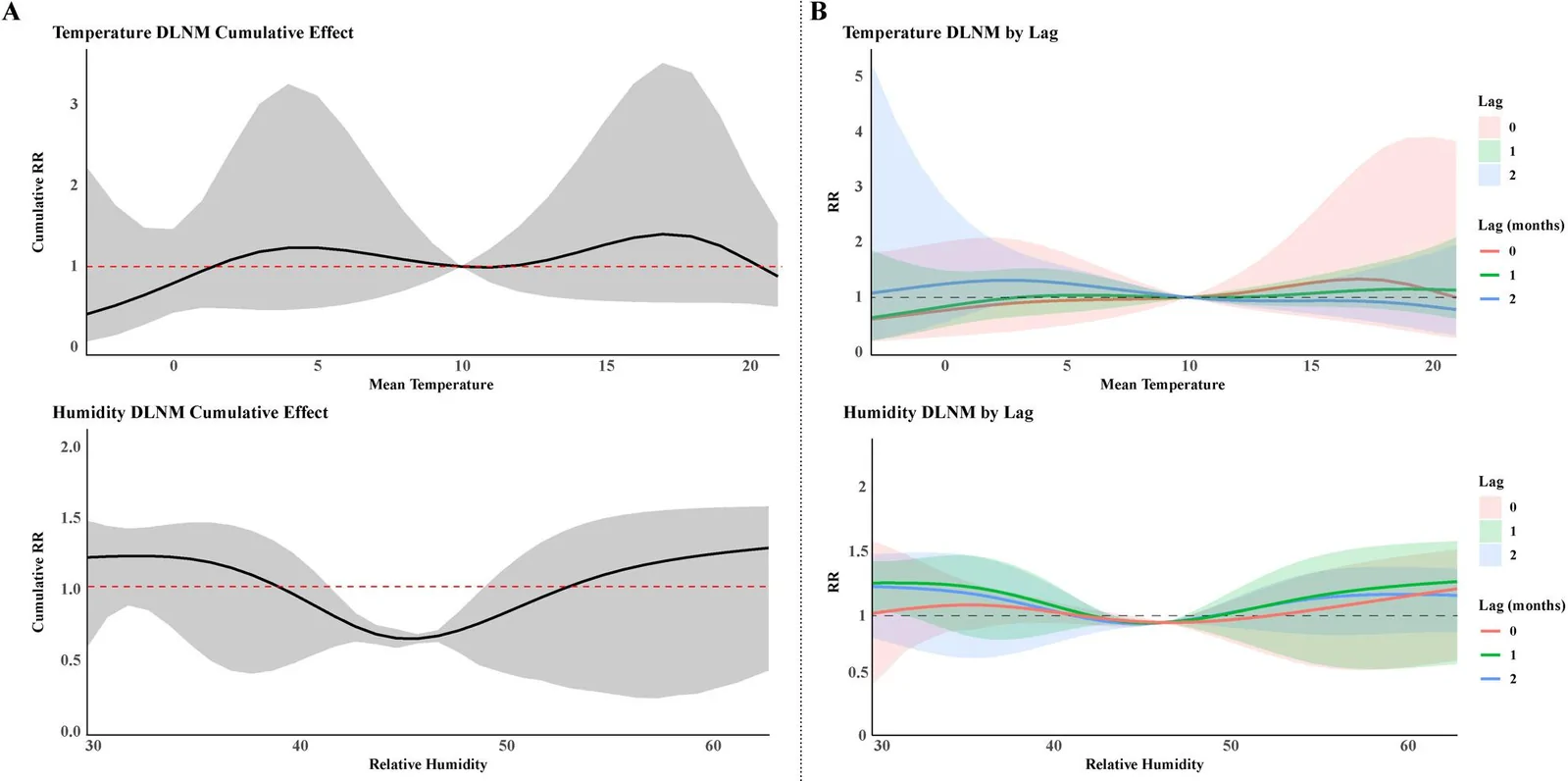
Distributed lag nonlinear model (DLNM) illustrating the relationships of temperature and relative humidity with the risk of respiratory failure in critically ill patients. **A** Cumulative effects of mean temperature and mean relative humidity on the risk of respiratory failure. **B** Lagged effects of mean temperature and mean relative humidity on the risk of respiratory failure across different lag periods. Shaded areas represent 95% confidence intervals

DLNM further elucidated the complex relationships between meteorological factors and the risk of respiratory failure in critically ill patients. Cumulative effect analysis indicated that both mean temperature and mean relative humidity exhibited nonlinear associations with risk (Fig. 4A). The cumulative relative risk (RR) of respiratory failure peaked at approximately 5 °C and 17 °C, suggesting a substantially increased risk of respiratory failure at these temperature levels. Similarly, mean relative humidity below 39% or above 53% was associated with elevated risk. Lag effect analysis further revealed the temporal dynamics of these influences (Fig. 4B). In the low-temperature range (0–10 °C), the RR at a 2-month lag exceeded 1, indicating a delayed effect of low temperatures. In the moderate-temperature range (10–20 °C), the elevated risk was primarily observed in the current month and gradually attenuated thereafter. Regarding relative humidity, when the monthly mean exceeded 52% or fell below 41%, the RR was greater than 1, with the risk effect at a 1-month lag stronger than at a 2-month lag, and the affected period narrowed over time.

**Fig. 5:**
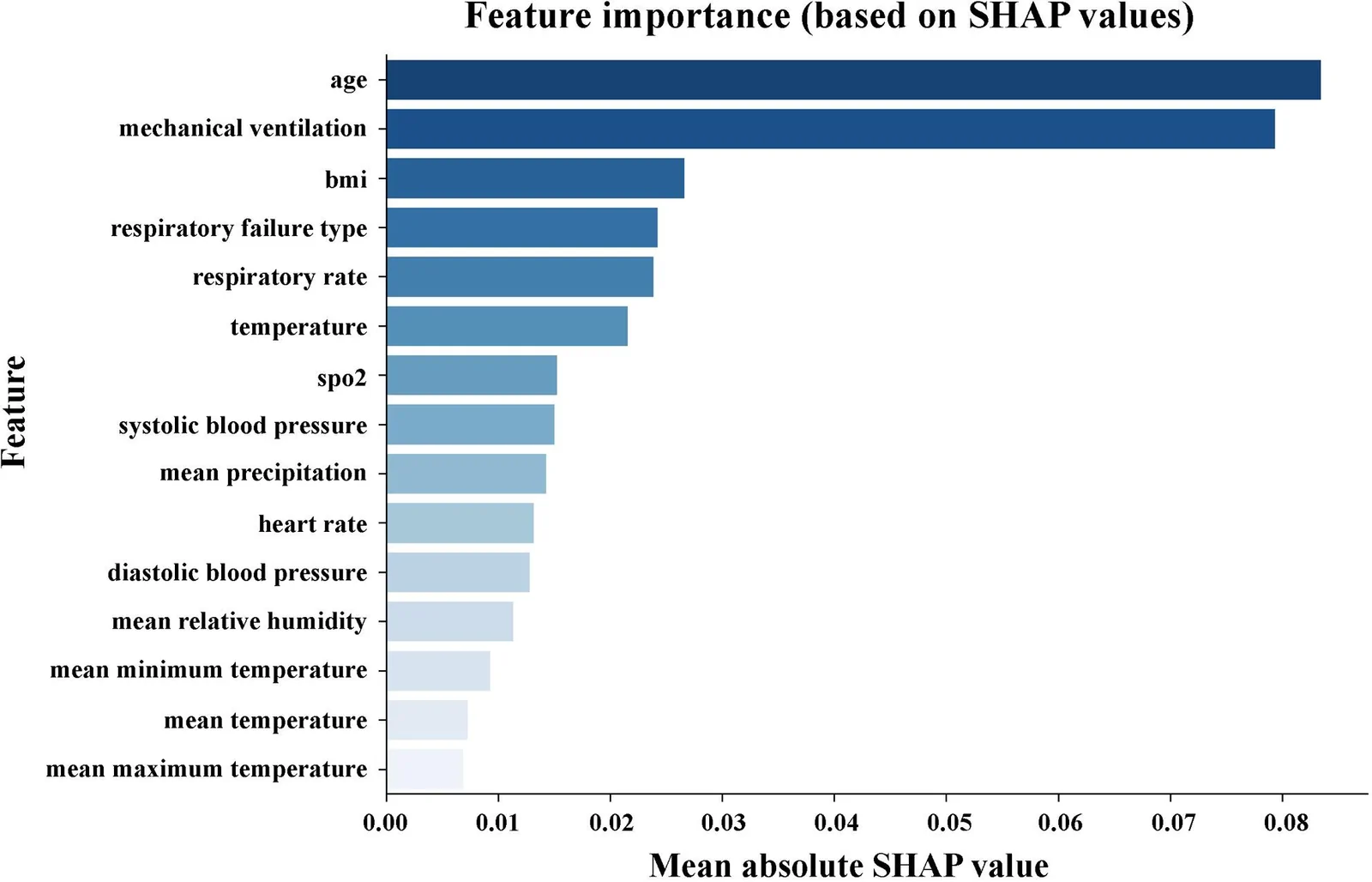
Relative importance of features in predicting patient mortality. The bar plot is ranked by mean absolute SHapley Additive exPlanations (SHAP) values calculated across testing-set observations and displays the top 15 variables contributing to death risk

**Fig. 6:**
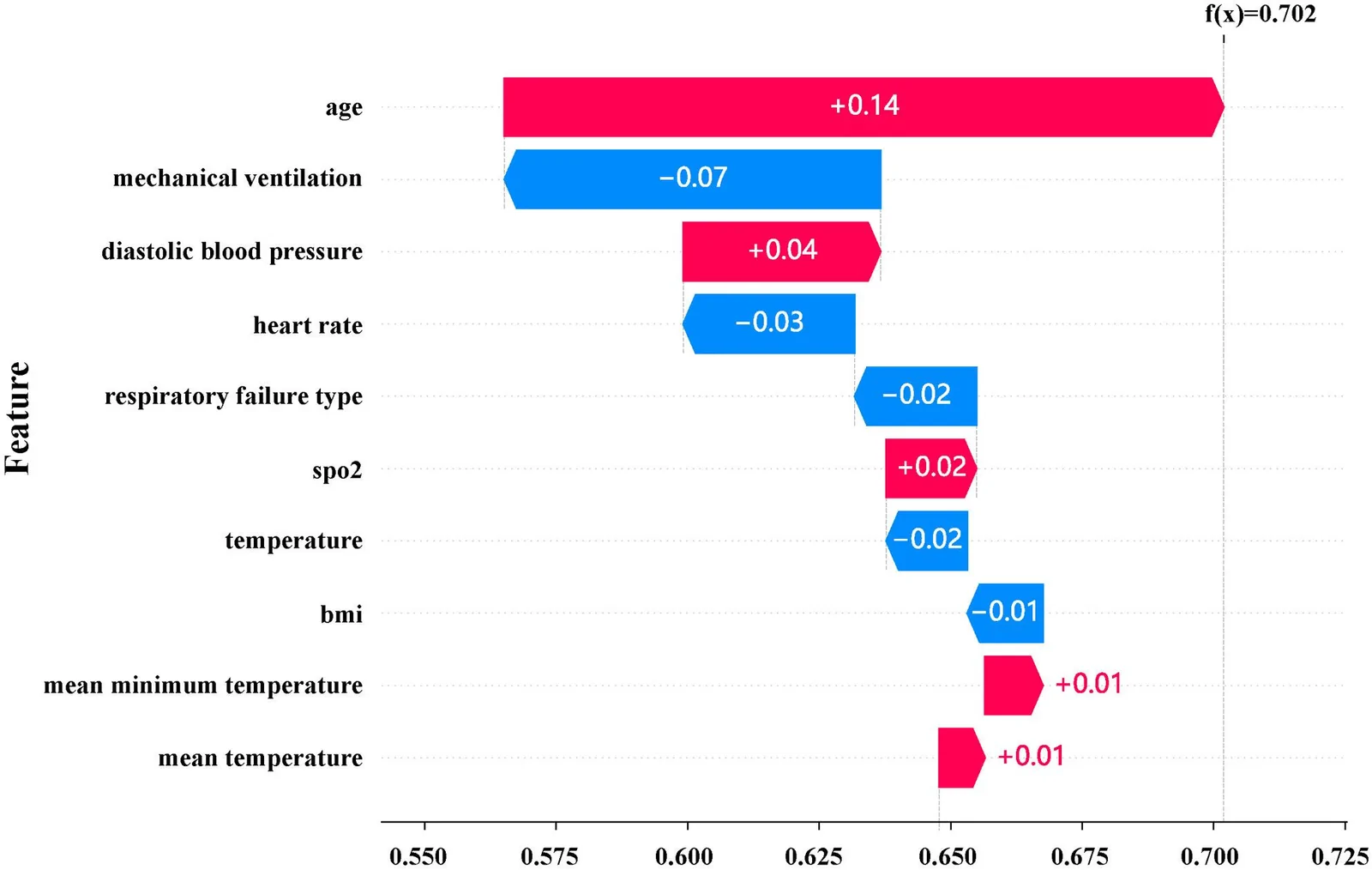
Waterfall plot showing feature contributions to the model-predicted mortality risk for an individual patient. Red indicates contributions that increase the model output relative to the baseline prediction, whereas blue indicates contributions that decrease it

SHAP analysis based on the random forest model indicated that the primary predictors of patient mortality were age and mechanical ventilation, with mean absolute SHAP values of 0.083 and 0.079, respectively, substantially higher than other variables. Body mass index (BMI), respiratory failure subtype, and respiratory rate also contributed notably to mortality risk (mean absolute SHAP values 0.027 − 0.024). Body temperature, oxygen saturation, blood pressure, and certain meteorological factors (e.g., mean precipitation and mean temperature) had moderate contributions, whereas sex and season of admission had minimal impact (mean absolute SHAP values < 0.005). A bar plot visually depicted the relative importance of each variable for overall predictions (Fig. 5). For the individual example presented in Fig. 6, age made the largest positive contribution to the predicted mortality risk, whereas the mechanical ventilation feature made the largest negative contribution; the remaining features showed smaller contributions in either direction.

## Discussion

In this 36-month multicenter retrospective study, conducted in the EICUs of two tertiary hospitals in Shijiazhuang, a representative city in northern China, a total of 1,837 critically ill patients with respiratory failure were included. Several key findings emerged. First, regarding baseline characteristics, patients with hypercapnic respiratory failure accounted for the largest proportion, nearly all patients received mechanical ventilation, and the in-hospital mortality exceeded 50%. Second, patient admissions exhibited significant seasonal variation, with the highest number of cases in winter and the lowest in summer. Third, meteorological factors, particularly temperature and relative humidity, were nonlinearly and lag-dependently associated with the incidence of respiratory failure. Finally, machine learning-based SHAP analysis indicated that age and mechanical ventilation were the primary determinants of mortality risk, followed by BMI, respiratory failure phenotype, and respiratory rate.

Our findings are consistent with previous studies [23, 28], showing that winter and spring represent high-risk seasons for the incidence and mortality of respiratory diseases, which may be closely related to low-temperature exposure, increased respiratory infections, and acute exacerbations of chronic cardiopulmonary diseases. The predominance of hypercapnic respiratory failure (64.07%) may reflect, among other factors, a substantial burden of chronic airway disease in this older population, given the high prevalence of chronic obstructive pulmonary disease (COPD) in China [29]. However, respiratory failure phenotype does not establish the primary etiology, and the present dataset cannot determine whether the winter peak was driven by acute exacerbations of COPD, pneumonia, acute heart failure, or overlapping conditions. Accordingly, the observed seasonal pattern should be interpreted at the syndrome level rather than as evidence for a specific etiologic subgroup. We observed significant lagged effects of meteorological factors on the occurrence of respiratory failure, with the strongest effect of temperature at a 1-month lag, suggesting that the impact of declining temperature on individual respiratory systems is not immediate but may increase disease risk after several weeks through cumulative or delayed triggering mechanisms. The immediate negative correlation between precipitation and case numbers indicates that short-term environmental changes may directly alter exposure levels in susceptible populations via mechanisms such as humidity modulation and pollutant deposition. The bidirectional effect of relative humidity is biologically plausible, presenting a U-shaped risk profile: excessively low humidity can dry the respiratory mucosa and enhance viral aerosol transmission, whereas excessively high humidity promotes the proliferation of molds and dust mites, with both extremes acting as potent triggers for respiratory disease exacerbations. These observations are in line with prior ecological studies linking climate variation to hospitalizations for respiratory diseases [30, 31].

Building on this, further analyses using GAM and DLNM revealed more complex nonlinear relationships and temporal dynamics between meteorological factors and the risk of respiratory failure. We found that temperature around 0°C and 20°C was associated with increased risk, while relative humidity at extreme levels (< 39% or > 53%) similarly elevated the risk of respiratory failure. These findings suggest that the effects of climatic factors on respiratory failure are not simply unidirectional or linear, but may involve a dual mechanism encompassing physiological stress responses, immune modulation, and pathogen survival environments [32]. Furthermore, the assessment of ‘cumulative effects’ in our DLNM analysis is a well-established methodological approach for capturing the net health burden of environmental exposures over a specific time window [33]. From a biological perspective, unlike immediate acute triggers, prolonged exposure to adverse meteorological conditions—such as sustained low temperatures—induces a state of continuous physiological stress and chronic inflammation in the airways [34]. This cumulative ‘wear-and-tear’ process leads to the progressive impairment of mucociliary clearance and the gradual depletion of the already limited cardiopulmonary reserves in elderly patients with pre-existing chronic respiratory diseases [35]. Consequently, the clinical manifestation of respiratory failure is often the threshold-crossing event of this accumulated physiological strain rather than the result of a single-day fluctuation. Results from the DLNM further highlighted the temporal characteristics of these effects: the risk associated with low-temperature exposure often manifests after a delay of several weeks, whereas the influence of relative humidity is primarily short-term. This observation aligns with our findings from the lagged correlation analysis, indicating that some climatic factors may affect respiratory health through a “delayed triggering” mechanism, while others exert more immediate effects. Our study not only validates previous findings on the relationship between meteorological conditions and respiratory disease risk [36, 37], but also expands the understanding of their action patterns and underlying mechanisms, providing a basis for establishing seasonal risk warning systems and targeted public health interventions in North China or other regions with similar climatic characteristics.

SHAP analysis revealed the determinants of prognostic risk at the individual level. Age and mechanical ventilation emerged as the most important predictors, consistent with extensive prior evidence, indicating that older patients and those requiring respiratory support have the poorest outcomes [38, 39]. BMI, respiratory failure phenotype, and respiratory rate also showed relatively high contributions, reflecting the importance of nutritional status, disease phenotype, and respiratory load in determining patient outcomes [40, 41]. In the complementary logistic regression analysis, a higher CCI remained associated with increased in-hospital mortality after adjustment for age, sex, respiratory failure phenotype, and mechanical ventilation status, indicating that chronic comorbidity burden was associated with prognosis in this cohort. SHAP waterfall plots visually illustrate the direction and magnitude of feature contributions to individual predictions, thereby complementing the population-level analysis. Notably, although meteorological variables were significantly associated with the occurrence of respiratory failure in ecological analyses [42–44], their contribution to individualized mortality prediction was relatively limited. This finding suggests that environmental exposures primarily influence population-level disease incidence and acute exacerbation risk, whereas once patients enter a critical state, clinical and physiological variables (e.g., age, mechanical ventilation) play a more decisive role in short-term prognosis. Similar conclusions have been reported previously: large-scale epidemiological studies have highlighted the contribution of meteorological factors to acute events and hospitalization rates in chronic diseases [28, 45], whereas prognostic models in critical care are predominantly driven by patient characteristics and organ function indicators [46]. Therefore, this study highlights the discrepancy between population-level environmental exposure and individual clinical prognosis, and suggests that future model development should integrate both ecological and clinical variables to achieve comprehensive risk management from population prevention to individualized treatment.

The main novelty of this study lies in the integration of multicenter critical care clinical data with meteorological and ecological data, systematically revealing for the first time the nonlinear and lagged relationships between meteorological factors, such as temperature and relative humidity, and critically ill patients with respiratory failure. At the individual level, SHAP analysis was used to elucidate the primary determinants of mortality risk. This approach not only identifies high-risk periods at the population level, providing a basis for seasonal early warning and allocation of critical care resources, but also highlights key clinical features at the individual level, offering guidance for precise prognostic assessment and treatment decisions. However, several limitations should be acknowledged. First, the retrospective study design restricts causal inference, and the observed associations, particularly those involving treatment-related variables, may be influenced by underlying disease severity, treatment selection, unmeasured confounding, and potential reverse causality [47, 48]. Second, meteorological data were derived from regional mean values and may not precisely represent individual patient exposures. Furthermore, meteorological variables such as temperature and precipitation exhibit strong seasonal collinearity, representing inherent residual confounding in ecological studies. In addition, complete district-level air-pollution data matched to both hospital areas throughout the study period could not be obtained; consequently, the models were not adjusted for particulate matter or gaseous pollutants. Because ambient air pollution may covary with meteorological conditions and influence respiratory morbidity, residual confounding by air pollution cannot be excluded [49]. Other contextual factors, including land-use patterns and socioeconomic conditions, were also unavailable [50, 51]. Therefore, the observed associations with meteorological factors should not be interpreted as effects that are independent of air pollution or other unmeasured environmental factors. Third, the retrospective database did not contain a prespecified variable assigning a single primary etiology of respiratory failure. Although chronic comorbidity burden was summarized using the Charlson Comorbidity Index, pneumonia and non-infectious conditions could coexist, and routine admission diagnoses did not permit reliable post hoc attribution of a single primary cause. Consequently, etiology-specific analyses of seasonal patterns and mortality could not be performed. COVID-19 status was not included in the original data-extraction framework, precluding reliable estimation of the proportion of patients with COVID-19. Future prospective studies should record primary and contributing etiologies using standardized diagnostic criteria. In addition, SOFA was not included in the prespecified data-collection framework, which precluded assessment of its association with mortality and direct comparison with the present prognostic analysis. Finally, although SHAP analysis enhances model interpretability, its external applicability to other regions or populations requires further validation [52, 53]. Future studies should incorporate data from additional centers to expand the analysis, thereby improving the generalizability and applicability of the findings.

## Conclusion

In summary, this study provides the first systematic analysis of critically ill patients with respiratory failure based on multicenter retrospective data from a representative city in North China, evaluating the impact of meteorological factors on disease occurrence and prognosis. Our findings demonstrate that temperature and relative humidity are significantly associated with the incidence of respiratory failure in a nonlinear and lagged manner, with winter and spring representing high-risk periods. At the individual level, age, mechanical ventilation, BMI, respiratory failure phenotype, and respiratory rate were identified as the primary predictors of mortality risk. By integrating ecological exposure data with clinical characteristics, this study elucidates population-level environmental risks while highlighting key prognostic factors at the individual level, providing a scientific basis for regional seasonal risk warning, critical care resource optimization, and precision clinical decision-making. Moreover, our results suggest that future risk models should consider the interplay between environmental exposures and individual clinical variables to enable comprehensive risk management from public health interventions to personalized treatment. Further multicenter and multi-regional studies are warranted to validate the external applicability of these findings and to explore additional potential environmental and socioeconomic determinants of disease occurrence and outcomes in critically ill patients with respiratory failure.

## Supplementary Information

Supplementary Material 1.

Supplementary Material 2.

Supplementary Material 3.

Supplementary Material 4.

## Data Availability

The de-identified clinical data that support the findings of this study are not publicly available because they contain sensitive patient information and are subject to institutional and ethical restrictions. Requests for access may be directed to the corresponding author and will be considered subject to approval by the participating institutions and the ethics committee. The meteorological data are available from the National Meteorological Science Data Center.
