# Supplementary material for: Seasonal dynamics and prognostic determinants of respiratory failure in critically ill adults: a multicenter retrospective observational study in Northern China
Source: BMC Pulm Med. 2026 Aug 18;26:514. doi: 10.1186/s12890-026-04576-6 (PMC13723126; doi:10.1186/s12890-026-04576-6)
Supplement: Supplementary file 1 [file 12890_2026_4576_MOESM2_ESM.docx]

**Supplementary Table 1. Pre-imputation missingness and handling of clinical variables used in the mortality prediction model.**

BMI, body mass index; MICE, multiple imputation by chained equations. Weight and height were used to calculate BMI. Variables with ≥30% missingness were individually assessed for clinical relevance before retention; variables with <30% missingness were imputed using MICE.

| **Variable** | **Missingness (%)** | **Handling** |
| --- | --- | --- |
| Weight (for BMI calculation) | 1.09 | MICE |
| Height (for BMI calculation) | 38.22 | Assessed for clinical relevance and retained |
| Heart rate | 0.01 | MICE |
| Systolic blood pressure | 1.66 | MICE |
| Diastolic blood pressure | 1.66 | MICE |
| Respiratory rate | 0.05 | MICE |
| Oxygen saturation (SpO₂) | 0.11 | MICE |
| Temperature | 2.52 | MICE |
