# Supplementary material for: Seasonal dynamics and prognostic determinants of respiratory failure in critically ill adults: a multicenter retrospective observational study in Northern China
Source: BMC Pulm Med. 2026 Aug 18;26:514. doi: 10.1186/s12890-026-04576-6 (PMC13723126; doi:10.1186/s12890-026-04576-6)
Supplement: Supplementary file 2 [file 12890_2026_4576_MOESM3_ESM.docx]

**Supplementary Table 2. Univariable and multivariable logistic regression analyses of factors associated with in-hospital mortality.**

The original CCI without age adjustment was used. Female sex, hypoxemic respiratory failure, and no mechanical ventilation were used as the respective reference categories. The multivariable model included all variables shown in the table.

| **Variable** | **Univariable OR (95% CI)** | **P value** | **Adjusted OR (95% CI)** | **P value** |
| --- | --- | --- | --- | --- |
| CCI, per 1-point increase | 1.30 (1.26–1.35) | <0.001 | 1.26 (1.20–1.32) | <0.001 |
| Age, per 1-year increase | 1.04 (1.03–1.04) | <0.001 | 1.01 (1.00–1.02) | 0.018 |
| Male sex | 0.82 (0.68–0.98) | 0.034 | 0.80 (0.66–0.98) | 0.029 |
| Hypercapnic vs hypoxemic RF | 0.61 (0.50–0.74) | <0.001 | 0.63 (0.51–0.77) | <0.001 |
| Mixed vs hypoxemic RF | 0.61 (0.30–1.23) | 0.168 | 0.62 (0.29–1.33) | 0.223 |
| Mechanical ventilation | 1.09 (0.48–2.49) | 0.835 | 1.65 (0.67–4.02) | 0.275 |
