# Supplementary material for: Seasonal dynamics and prognostic determinants of respiratory failure in critically ill adults: a multicenter retrospective observational study in Northern China
Source: BMC Pulm Med. 2026 Aug 18;26:514. doi: 10.1186/s12890-026-04576-6 (PMC13723126; doi:10.1186/s12890-026-04576-6)
Supplement: Supplementary file 3 [file 12890_2026_4576_MOESM4_ESM.docx]

**Supplementary Table 3.** **Pearson correlations between meteorological variables and monthly cases (–2 to +2 month lag).**

| Variable | Lag month | Correlation coefficient | P-value | No. of cases |
| --- | --- | --- | --- | --- |
| Mean temperature | -2 | -0.0996 | 0.575 | 34 |
| Mean temperature | -1 | -0.5591 | 0.000 | 35 |
| Mean temperature | 0 | -0.8467 | 0.000 | 36 |
| Mean temperature | 1 | -0.8872 | 0.000 | 35 |
| Mean temperature | 2 | -0.6714 | 0.000 | 34 |
| Mean minimum temperature | -2 | -0.1643 | 0.353 | 34 |
| Mean minimum temperature | -1 | -0.6128 | 0.000 | 35 |
| Mean minimum temperature | 0 | -0.8754 | 0.000 | 36 |
| Mean minimum temperature | 1 | -0.8740 | 0.000 | 35 |
| Mean minimum temperature | 2 | -0.6265 | 0.000 | 34 |
| Mean maximum temperature | -2 | -0.0665 | 0.709 | 34 |
| Mean maximum temperature | -1 | -0.5250 | 0.001 | 35 |
| Mean maximum temperature | 0 | -0.8240 | 0.000 | 36 |
| Mean maximum temperature | 1 | -0.8901 | 0.000 | 35 |
| Mean maximum temperature | 2 | -0.6924 | 0.000 | 34 |
| Mean precipitation | -2 | -0.1732 | 0.327 | 34 |
| Mean precipitation | -1 | -0.4372 | 0.009 | 35 |
| Mean precipitation | 0 | -0.6519 | 0.000 | 36 |
| Mean precipitation | 1 | -0.6357 | 0.000 | 35 |
| Mean precipitation | 2 | -0.2959 | 0.089 | 34 |
| Mean relative humidity | -2 | -0.6950 | 0.000 | 34 |
| Mean relative humidity | -1 | -0.7778 | 0.000 | 35 |
| Mean relative humidity | 0 | -0.6514 | 0.000 | 36 |
| Mean relative humidity | 1 | -0.2045 | 0.239 | 35 |
| Mean relative humidity | 2 | 0.2242 | 0.202 | 34 |
